# Algorithms in Allergy: Molecular Allergology in the Context of Animal Allergy

**DOI:** 10.1111/all.70234

**Published:** 2026-01-26

**Authors:** Christiane Hilger, Bulent E. Sekerel, Marianne van Hage, Wolfgang Hemmer, Jon R. Konradsen

**Affiliations:** ^1^ Department of Infection & Immunity Luxembourg Institute of Health Esch‐sur‐Alzette Luxembourg; ^2^ Pediatric Allergy and Asthma Division, School of Medicine Hacettepe University Ankara Türkiye; ^3^ Division of Immunology and Respiratory Medicine, Department of Medicine Solna Karolinska Institutet Stockholm Sweden; ^4^ Center for Molecular Medicine Karolinska University Hospital Stockholm Sweden; ^5^ Department of Clinical Immunology and Transfusion Medicine Karolinska University Hospital Stockholm Sweden; ^6^ Floridsdorf Allergy Center Vienna Austria; ^7^ Astrid Lindgren Children's Hospital, Karolinska University Hospital Stockholm Sweden; ^8^ Department of Women's and Children's Health Karolinska Institutet Stockholm Sweden

**Keywords:** allergens and epitopes, allergy diagnosis, IgE, immunotherapy clinical, precision medicine

Allergies to furry animals are common, with symptoms ranging from mild rhinoconjunctivitis to severe asthma [1]. In the US and European adult population, sensitization rates to animal dander are reported at 10%–14% [2]. However, symptoms may occur without detectable sensitization, and sensitization may exist without clear symptoms, complicating diagnosis and clinical decision‐making. Even when sensitization and symptoms coexist, identifying the primary sensitization source remains crucial. Traditional extract‐based diagnostics are limited by variable allergen concentrations and frequent cross‐reactivity. Molecular allergy diagnostics (MA) enable precise patient profiling by detecting IgE to individual allergen molecules, thereby improving diagnostic accuracy and guiding personalized care.

Animal dander extracts contain multiple allergens, some serving as marker allergens indicating genuine sensitization to a specific animal, others being cross‐reactive [3], Figure 1. Marker allergens include secretoglobins (Fel d 1, Ory c 3), lipocalins (Can f 2, Can f 4; Ory c 1, Ory c 2; Cav p 1, Cav p 2, Cav p 3; Phod s 1; Mes a 1; Bos d 2), latherins (Fel d 8, Equ c 4), and the kallikrein Can f 5 [1]. In furry animals, cross‐reactive allergens fall into three main families: serum albumins, lipocalins, and cystatins. Serum albumins and cystatins share high sequence identity within their families, whereas lipocalins show more variability, with sequence identities ranging from low (20%–30%) to moderate and high (> 60%) [4]. Dog lipocalins Can f 2 and Can f 4 are marker allergens, whereas Can f 1 shares 62% identity with Fel d 7, and Can f 6 shows up to 67% identity with cat, horse, rabbit, guinea pig, rat, and mouse lipocalins [1]. Phylogenetically, these lipocalins form two well‐separated groups, which we propose labeling “Can f 1‐like” and “Fel d 4‐like” lipocalins to facilitate recognition. Sequence identity between these groups is low, and IgE cross‐reactivity has not been observed. Can f 7, a Niemann Pick type C2 (NPC2) protein, is likely to be cross‐reactive as it is a highly conserved mammalian protein.

**FIGURE 1:**
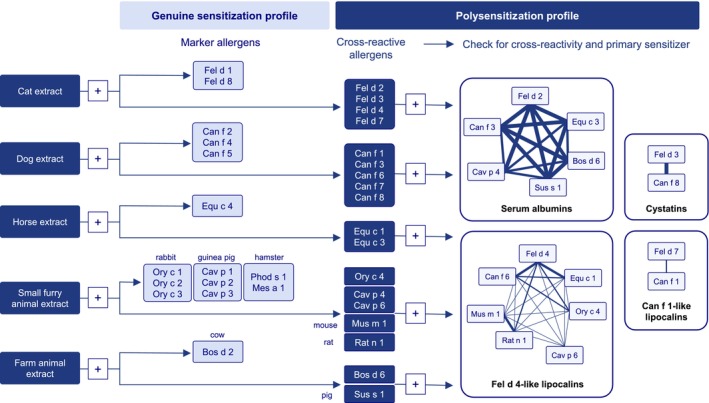
Diagnostic algorithm for molecular diagnosis in animal allergic patients. Respiratory allergens from furry animals that have been approved by the WHO/IUIS Allergen Nomenclature Sub‐Committee https://www.allergen.org are classified as marker allergens or cross‐reactive allergens. Bos d 6 and Sus s 1, although being food allergens, are included as they play an important role in clinical cross‐reactivity to animal dander. Not all molecular allergens listed are already available for in vitro sIgE quantification. Currently available extracts from farm animals include cow, goat, sheep, and swine; small furry animal extracts are available for chinchilla, ferret, gerbil, guinea pig, hamster, mink, mouse, rabbit, and rat. Many allergens from those sources remain uncharacterized. The broadness of the connecting lines at the right shows levels of sequence identity from lowest (thinnest line) to highest (thickest line) 50% (45–55), 60% (56–65), 70% (66–75), and > 75% between individual allergens.

Clinically, Fel d 1 is a highly specific marker for cat allergy, with IgE prevalence exceeding 90% in cat‐allergic patients [1]. Sensitization to specific cat allergen molecules—particularly Fel d 1, Fel d 2, and Fel d 4—is more strongly associated with asthma, wheeze, and type 2 inflammation than IgE to cat extract alone. Higher IgE levels to multiple cat allergens also correlate with increased disease severity [1].

Dog‐allergic patients frequently show polysensitization, with Can f 5, Can f 1, and Can f 4 being the most commonly recognized allergens [5, 6]. Polysensitization to multiple dog molecules—especially lipocalins such as Can f 2, Can f 4, and Can f 6—better predicts dog allergy and asthma severity than sensitization to dog dander extract alone [5]. In contrast, monosensitization to the male dog allergen Can f 5 is typically associated with mild symptoms and negative provocation tests, suggesting that individuals sensitized only to Can f 5 may tolerate exposure to female or neutered dogs. Adults and children with suspected allergic rhinitis or asthma often show complex sensitization patterns to animal allergens. Polysensitization involving cross‐reactive lipocalins (Fel d 4/Can f 6/Equ c 1 and Fel d 7/Can f 1) is common and linked to allergy, and the allergen with the highest specific IgE level typically indicates the primary sensitizer [6].

In clinical practice, the initial assessment of patients presenting with indications for animal allergy testing should be extract‐based testing, using either serum‐specific IgE measurement towards allergen extracts or skin prick tests (SPT), Figure 2. Subsequent steps depend on the clinical context and results from extract‐based tests. In many cases, MA can be considered. The first priority in MA is to assess sensitization to marker allergens, as a positive result suggests genuine allergy to the corresponding animal, provided that the clinical history is consistent with sensitization. If no marker allergens are detected, or if there is clinical suspicion of cross‐ or co‐sensitization, evaluation of cross‐reactive allergens should be pursued. For patients in whom allergy is confirmed by history and sensitization, symptomatic management and allergen avoidance should be implemented. If these interventions are insufficient, allergen immunotherapy is indicated.

**FIGURE 2:**
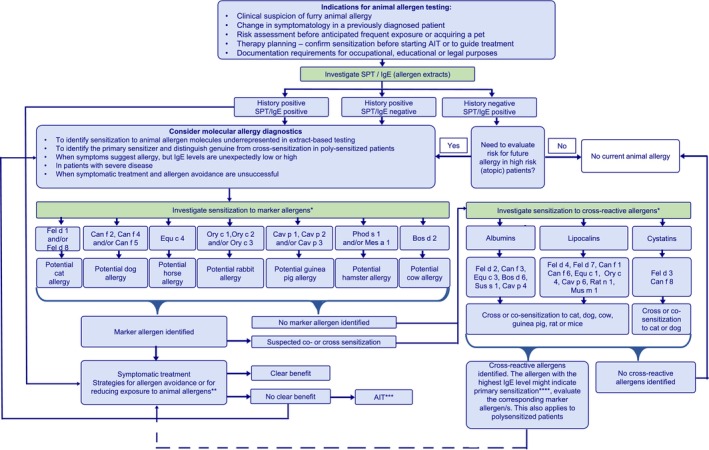
Clinical algorithm for diagnosis and treatment of patients with suspected animal allergy. *We recommend a stepwise approach for the interpretation of test results, but sampling for marker allergens and cross‐reactive allergens can be done simultaneously. Multiplex analysis is advantageous for a comprehensive assessment. **No single measure is fully effective; combined strategies are needed: HEPA vacuuming/air filtration, washing the animal, removing carpets, restricting bedroom access, frequent ventilation, and neutering. ***Allergen immunotherapy may be considered for a proven animal allergy if symptoms continue despite allergen avoidance. The allergen molecule content (standardization) of the immunotherapy product (extract) should be a key factor in this decision. ****The accuracy of IgE quantification depends on the analytical sensitivity of the assay used.

Importantly, not all furry animal allergens have been identified, and particularly allergens from horse, ferret, or chinchilla may still be missed. The discovery of new allergens may refine current marker‐allergen classifications. Molecular allergology is rapidly advancing, and the continued availability of new molecules and in vitro IgE diagnostic platforms will further enable patient‐tailored diagnosis and therapy.

## Author Contributions

All authors contributed to conceptualizing the text and figures. C.H. and J.R.K. drafted the text and figures. All authors critically reviewed the text and figures for intellectual content. All authors approved the final manuscript and agreed to be accountable for the integrity of the work.

## Funding

C.H. was supported by the Luxembourg National Research Fund (FNR) (grant no 2021/16734157). M.v.H. was supported by the Swedish Heart‐Lung Foundation (grant number 20240563), the Region Stockholm (ALF project FoUI‐986234), The Swedish Asthma and Allergy Association's Research Foundation (grant number F2022‐0011), The Swedish Cancer and Allergy Foundation, The Hesselman Foundation. J.R.K. was supported by Region Stockholm (Högre Klinisk Forskare FoUI‐1002791), the Foundation “Frimurare Barnhuset in Stockholm,” and the Konsul Th. C. Bergh's Foundation (240018).

## Conflicts of Interest

C.H. reports speaker honorarium in the past 5 years from ALK‐Abello and Siemens Healthcare Diagnostics, outside the submitted work. B.E.S. declares no conflicts of interest. M.v.H. reports speaker honorarium from Thermo Fisher Scientific and ALK. W.H. reports speaker honorarium from ALK, outside the submitted work. J.R.K. reports advisory board fees from Novartis and ALK, nonfinancial support from Thermo Fisher Scientific and institutional fees from Regeneron Pharmaceuticals outside the submitted work.

## Data Availability

This manuscript presents a clinically oriented algorithm derived from previously published evidence; no original data were generated or analyzed.
